# Efficacy of *Brucella* Vaccines in Sheep: A Systematic Review and Meta-Analysis

**DOI:** 10.1155/2024/5524768

**Published:** 2024-07-26

**Authors:** Lian-Min Li, Wen-Tao Xiang, Ting Li, Mei-Mei Xiang, Fei Liu, Jian-Ming Li

**Affiliations:** ^1^College of Chinese Medicine Materials, Jilin Agricultural University, Changchun 130118, Jilin Province, China; ^2^College of Animal Science and Technology, Jilin Agricultural University, Changchun 130118, Jilin Province, China

## Abstract

**Background:**

Brucellosis is a major worldwide public health problem with economic and zoonotic implications. Despite the importance of vaccines in preventing brucellosis, no previous systematic evaluation of vaccination in sheep has been conducted.

**Materials and Methods:**

Articles were searched in databases such as PubMed, Science Direct, Cochrane, VIP, Wan Fang, and CNKI by screening the articles, and articles reporting *Brucella* vaccination in sheep were included in the study. Meta-analysis was performed using random effects models to calculate pooled risk ratios for vaccines and to calculate vaccine effectiveness.

**Results:**

A total of 2,605 articles were retrieved, and 17 articles were obtained through screening for analysis. The effectiveness of vaccination was 65% (RR = 0.35, 95% CI: 0.27–0.36; VE = 65%), with the M5 vaccine being significantly more effective at 84% (RR = 0.1587, 95% CI: 0.0256–0.9858; VE = 84%) than the other vaccines, and intramuscular injection could be the best route of immunization. Rev.1 was indicated for female sheep, especially for pregnant ewes (RR = 0.2016, 95% CI: 0.1139–0.3569; VE = 80%), and for reduced abortions (RR = 0.0978, 95% CI: 0.0459–0.2085).

**Conclusion:**

This meta-analysis was conducted to identify the relevant factors affecting vaccine efficacy. We recommend that sheep be inoculated intramuscularly with Rev.1, different inoculation protocols be adopted for sheep of different ages, and pregnant ewes be inoculated with Rev.1 to prevent abortion.

## 1. Introduction

Bacteria of the genus Brucella are members of the alpha-2 Proteobacteria and are parthenogenetic intracellular and Gram-negative bacteria [1]. In recent years, the number of new *Brucella* species has increased, and there are currently 12 *Brucella species*, three of which can cause severe brucellosis in humans [2]. Brucellosis, a globally occurring subacute or chronic zoonotic disease that infects cattle, sheep, goats, other ruminants, and pigs, is usually unnoticeable in the initial stages of infection, with abortion, placenta retention, uterine inflammation, and reduced milk production occurring in the later stages [3, 4, 5].

Sheep act as natural hosts for *Brucella*, and this disease can occur in most major sheep-rearing countries. Brucellosis can be sexually transmitted from infected sheep to other healthy sheep to infect the flock [6]. Outside of the breeding season, the disease is also transmitted directly between sheep via oral and mucosal routes. When sheep are infected, rams can cause testicular inflammation, arthritis, and other symptoms, and the main clinical phenomena in ewes are abortion and stillbirths [7]. In addition to adversely affecting the health of flocks, sheep brucellosis has significant economic consequences, including reduced conception rates, prolonged lambing periods, reduced marketability, and the loss of valuable genes from culled rams [8].

Because of the serious economic and medical consequences of brucellosis, efforts have been made to prevent infection through the use of vaccines [9]. In endemic areas, vaccination against the epidemic is the most economical preventive measure to control brucellosis [10]. The technology used for vaccine development can be divided into traditional vaccines and new vaccines. Traditional vaccines include inactivated vaccines and live attenuated vaccines, and their components generally include the whole pathogen [11]. Live attenuated vaccine strains are the most effective vaccines available for the control of brucellosis in animals and play an important role in the prevention and control of brucellosis in animals and humans [12, 13] because live attenuated vaccine strains have the same tissue tropism and cellular tropism as wild-type strains, as opposed to inactivated and subunit vaccines, and can induce immunity through humoral and cell-mediated responses, thus providing a greater level of protection [14, 15]. Rev.1 and M5 are widely used as commonly used live attenuated vaccines against brucellosis in small ruminants. In 1957, Rev.1 was obtained by successive passaging of virulent *B. melitensis* 6,056 on streptomycin-containing media and is used in several countries and regions [16]. In 1991, the Harbin Veterinary Medical Research Institute in China produced M5 from the potent strain *B. melitensis* M28 by acridine yellow treatment and sequential passaging in cultured chicken embryo fibroblasts, which is commonly used for the prevention of brucellosis in sheep and goats in China [17]. Both Rev.1 and M5 are produced by successive passages of weakened strong strains, which are simple to produce, but the molecular and physiological mechanisms underlying the loss of vaccine virulence are unknown [18, 19]. In addition, vaccination can cause abortions in pregnant animals, interfere with serological diagnosis, and make it impossible to distinguish between vaccine and natural infections, thus leading to misclassification of the source of infection.

The lack of available vaccines hampers the control of human brucellosis. Therefore, the control of animal brucellosis is the most effective way to prevent human infections [20]. This paper presents the first meta-analysis of the clinical phenomenon of brucellosis vaccination in sheep and the development of rational vaccination recommendations to prevent the spread of brucellosis in sheep better, thereby improving the health and safety of animals and related workers.

## 2. Materials and Methods

### 2.1. Search Strategy

We searched six databases, including Cochrane, Science Direct, PubMed, VIP, Wan Fang, and CNKI, for eligible studies published before January 2, 2024. The search strategy is detailed in *Supplementary Appendix 1*. The database search results were downloaded and put into EndNote (EndNote X9.3.3).

### 2.2. Inclusion and Exclusion Criteria

Inclusion criteria are as follows:

1. Studies using placebo or other vaccines as control or experimental groups.
2. This study reports the efficacy of a vaccine against brucellosis in sheep.
3. Articles published in English or Chinese.

Exclusion criteria are as follows:

1. Failure to conduct bacteriological attack experiments.
2. No control group.
3. Unable to download.
4. The experimental animal is not a sheep.
5. Repeated articles.

### 2.3. Data Extraction

We used EndNote X9.3.3 to manage records and filter and exclude duplicates. After article screening through inclusion and exclusion criteria, the following data were extracted from the included articles: first author, year of publication, geographic location, vaccine type, mode of immunization, dose of immunization, region, number of samples, sex, age at the time of vaccination, data on protection from clinical symptoms (abortive and bacteriological), data on protection from infections (abortive and bacteriological), strains of challenge infections, and doses of infections. The extracted information was exported to Microsoft® Excel® 2019 MSO (16.0.14228.20216) 32.

### 2.4. Statistical Methods

The quality of the included studies was assessed based on the criteria of the Grading of Recommendations Assessment, Development, and Evaluation (GRADE) method (*Supplementary table 2*) [21]. The included articles were analyzed in this study using R version 4.0.0. Combined risk ratios and 95% confidence intervals were calculated using a random effects meta-analysis model [22]. Vaccine effectiveness was calculated as (1 − risk ratio) [23]. The presence of publication bias was assessed using funnel plots and Egger's test, and *P* < 0.05 indicated statistical significance [24]. When the funnel plot was asymmetric, the shear method was used for correction [25]. The *I*^2^ test was used to assess the statistical heterogeneity between studies; the higher the *I*^2^ value was, the greater the heterogeneity [26].

## 3. Results

### 3.1. Selected Studies

**Figure 1:**
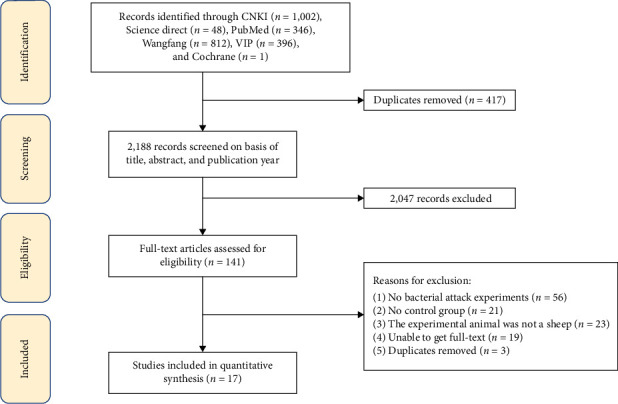
Flow diagram of the search and selection of eligible studies.

In this study, 2,605 studies were collected from six databases. First, duplicates were removed using EndNote. Second, articles were filtered by article title and abstract. Finally, 17 studies were subjected to data extraction based on the inclusion and exclusion criteria (Table 1 and Figure 1). The vaccine types included in the study were mainly live attenuated (M-111, Rev.1, S2, M5, and RB51) and inactivated (*B. ovis*).

### 3.2. Publication Bias and Risk of Bias Assessment

**Figure 2:**
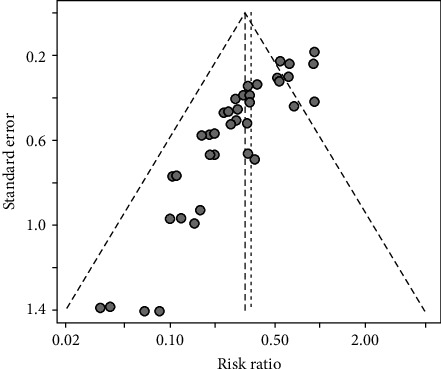
Funnel chart to detect publication bias.

**Figure 3:**
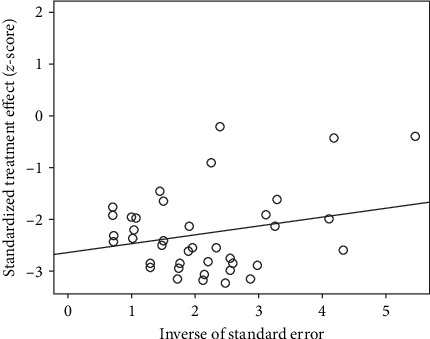
Egger detected publication bias in the study.

**Figure 4:**
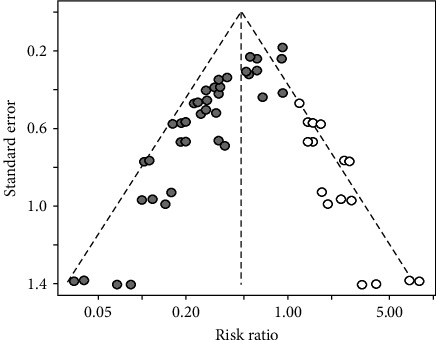
Trim-and-fill chart to detect research bias.

**Figure 5:**
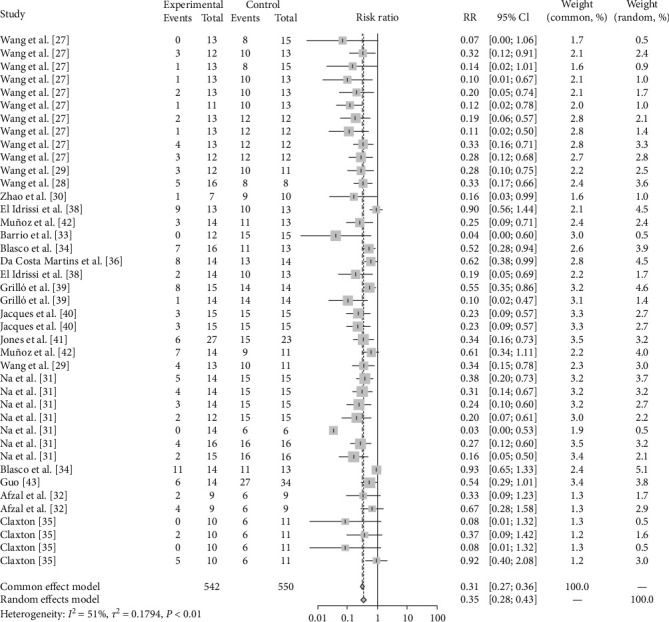
Forest plot showing heterogeneity between studies.

Publication bias was shown to exist in the tests by the funnel plot (Figure 2) and Egger (*P*-value <0.0001) (Figure 3 and *Supplementary table 2*), so the stability of the combined results was assessed by the trim-and-fill method, and the results showed good stability (Figure 4). The result of the sensitivity analysis is *I*^2^ = 51%, indicating heterogeneity in the meta-analysis, and the following analysis was carried out to determine the source of heterogeneity (Figure 5).

### 3.3. Vaccine-Related Outcomes

**Figure 6:**
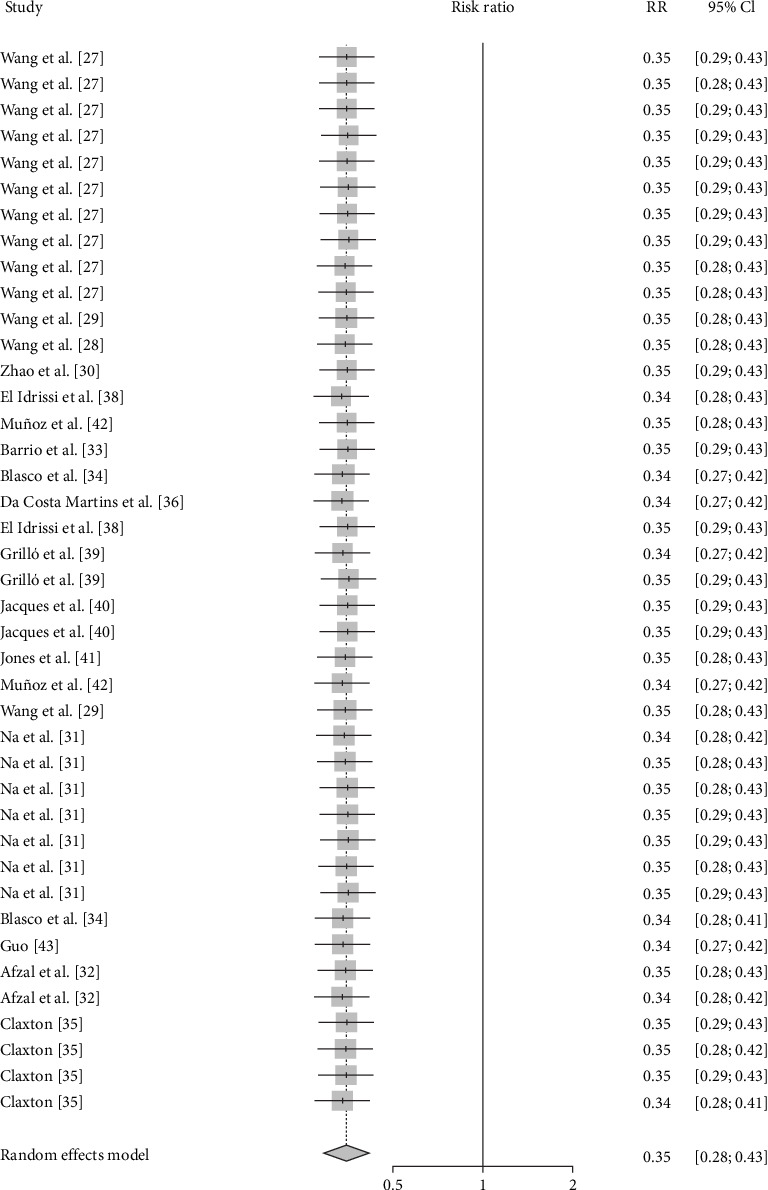
Forest plot of the pooled protective effect of vaccination against brucellosis infection after exposure to virulent *Brucella*.

**Figure 7:**
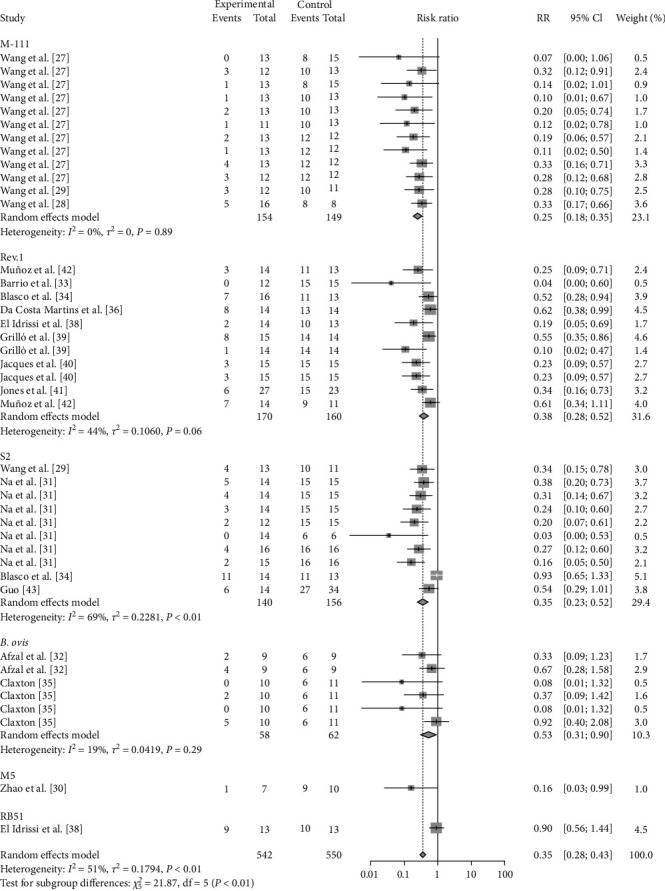
Forest plot of the protective effect of vaccination with different species against brucellosis infection after exposure to virulent *Brucella*.

The results of this study showed that a meta-analysis of all studies with sheep vaccination as an outcome indicated that the pooled efficacy of the vaccine was approximately 65% (RR = 0.35, 95% CI: 0.27–0.36; VE = 65%) (Figure 6). The effectiveness of the M5 vaccine was 84% (RR = 0.1587, 95% CI: 0.0256–0.9858; VE = 84%), which was significantly greater than that of the other vaccines (*P*=0.0401) (Table 2, Figure 7, and *Supplementary figure 3*).

**Figure 8:**
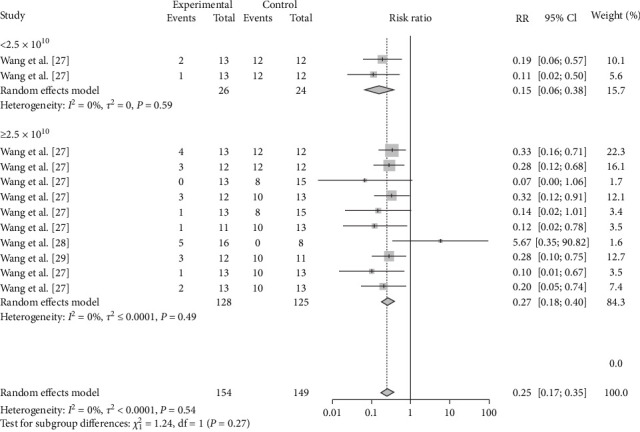
Forest plot of the protective effect of different doses of M-111 against brucellosis infection after exposure to virulent *Brucella*.

**Figure 9:**
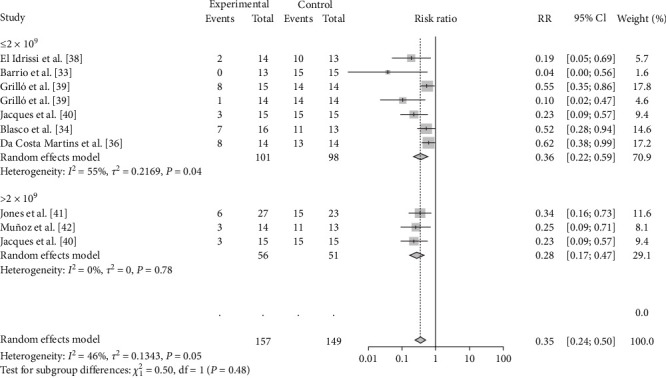
Forest plot of the protective effect of different doses of Rev.1 against brucellosis infection after exposure to virulent *Brucella*.

**Figure 10:**
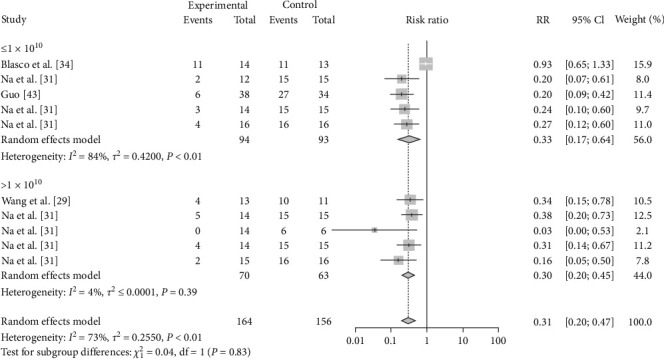
Forest plot of the protective effect of different doses of S2 inoculation against brucellosis infection after exposure to virulent *Brucella*.

**Figure 11:**
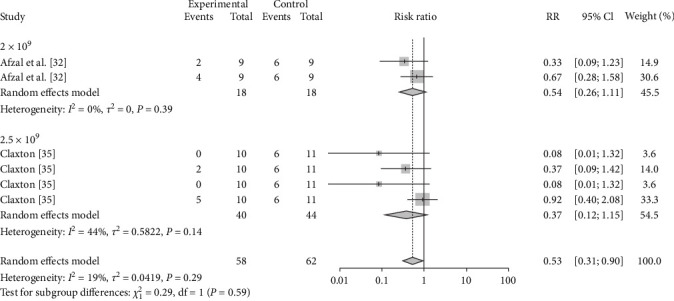
Forest plot of the protective effect of different doses of *B. ovis* against brucellosis infection after exposure to virulent *Brucella*.

The vaccination doses were analyzed to determine the optimal dose of each vaccine. The results showed that M-111 was more effective than ≥2.5 × 10^10^ (RR = 0.2692, 95% CI: 0.1826–0.3967; VE = 73%) at an inoculation dose <2.5 × 10^10^ (RR = 0.1545, 95% CI: 0.0629–0.3795; VE = 85%), with no significant difference (Table 3, Figure 8, and *Supplementary figure 3*). The Rev.1 vaccine was less effective than the >2 × 10^9^ vaccine (RR = 0.2790, 95% CI: 0.1673–0.4653; VE = 72%) at doses ≤2 × 10^9^ (RR = 0.3604, 95% CI: 0.2208–0.5882; VE = 64%), with no significant difference (Table 3, Figure 9, and *Supplementary figure 3*). The S2 vaccine was less effective than the >1 × 10^10^ vaccine (RR = 0.2999, 95% CI: 0.2016–0.4461; VE = 70%) at a vaccination dose ≤1 × 10^10^ (RR = 0.3260, 95% CI: 0.1671–0.6358; VE = 67%), with no significant difference (Table 3, Figure 10, and *Supplementary figure 3*). *B. ovis* was more effective at an inoculation dose of 2.5 × 10^9^ (RR = 0.3736, 95% CI: 0.1216–1.1482; VE = 63%) than at an inoculation dose of 2 × 10^9^ (RR = 0.5399, 95% CI: 0.2626–1.1101; VE = 46%), with no significant difference (Table 3, Figure 11, and *Supplementary figure 3*).

**Figure 12:**
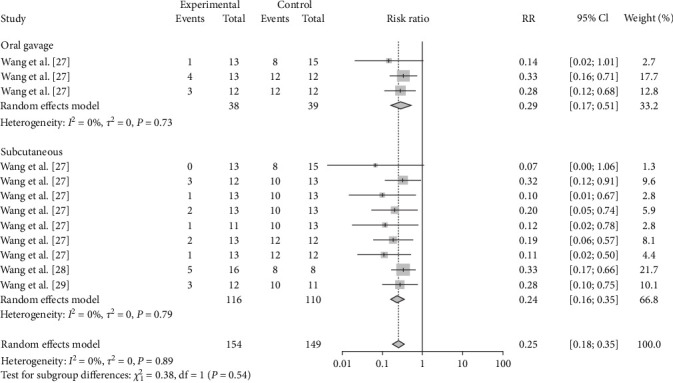
Forest plot of the protective effect of different inoculation modes of M-111 against brucellosis infection after exposure to virulent *Brucella*.

**Figure 13:**
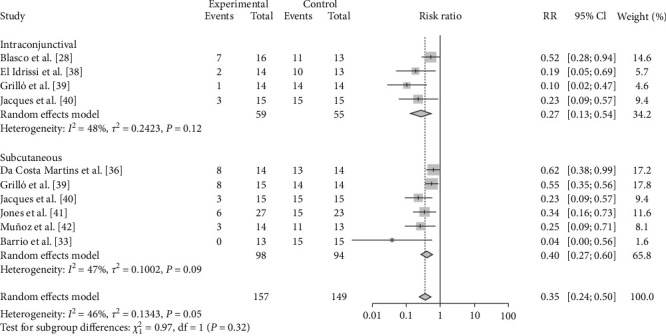
Forest plot of the protective effect of different inoculation modes of Rev.1 against brucellosis infection after exposure to virulent *Brucella*.

**Figure 14:**
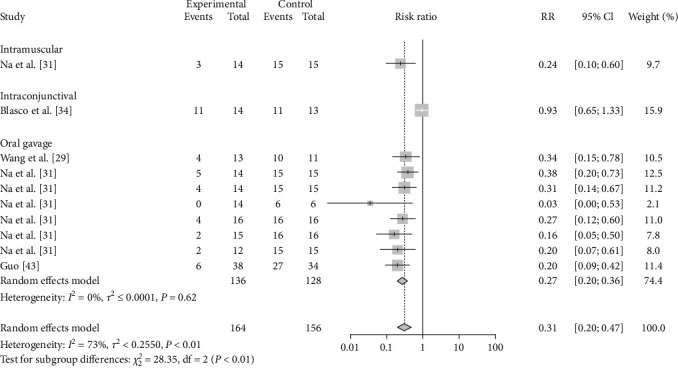
Forest plot of the protective effect of different inoculation modes of S2 against brucellosis infection after exposure to virulent *Brucella*.

As the route of vaccine immunization is an important factor affecting the effectiveness of vaccine immunization, we also analyzed the different routes of immunization for different vaccines. The results showed that subcutaneous M-111 vaccination (RR = 0.2355, 95% CI: 0.1596–0.3474; VE = 77%) was more effective than oral gavage (RR = 0.2913, 95% CI: 0.1678–0.5057; VE = 71%), with no significant difference (Table 4, Figure 12, and *Supplementary figure 3*). Rev.1 vaccination via intraconjunctival (RR = 0.2653, 95% CI: 0.1313–0.5361; VE = 73%) was more effective than subcutaneous (RR = 0.3993, 95% CI: 0.2654–0.3474; VE = 60%) vaccination, with no significant difference (Table 4, Figure 13, and *Supplementary figure 3*). Intramuscular S2 vaccination (RR = 0.2414, 95% CI: 0.0969–0.6012; VE = 76%) was significantly more effective than intraconjunctival vaccination and oral gavage vaccination (Table 4, Figure 14, and *Supplementary figure 3*).

### 3.4. Sheep-Related Results

**Figure 15:**
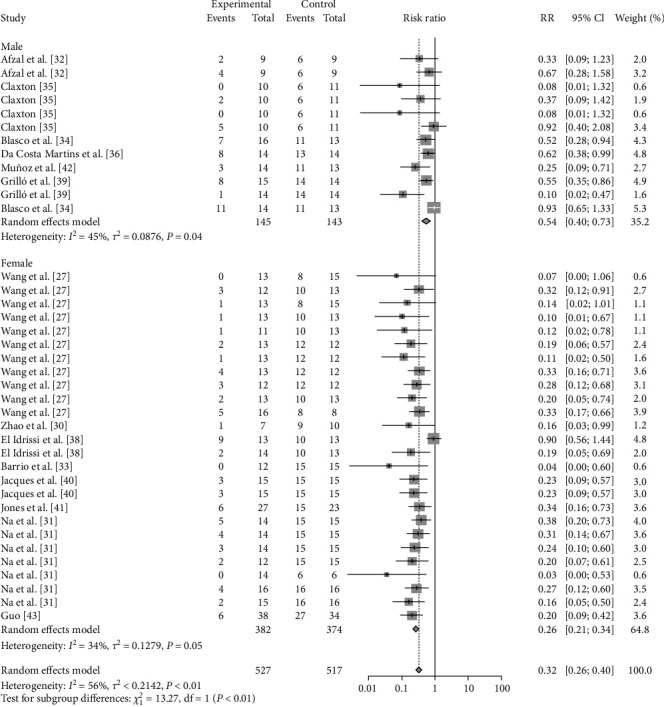
Forest plot of the protective effect of vaccination at different sexes against brucellosis infection after exposure to virulent *Brucella*.

**Figure 16:**
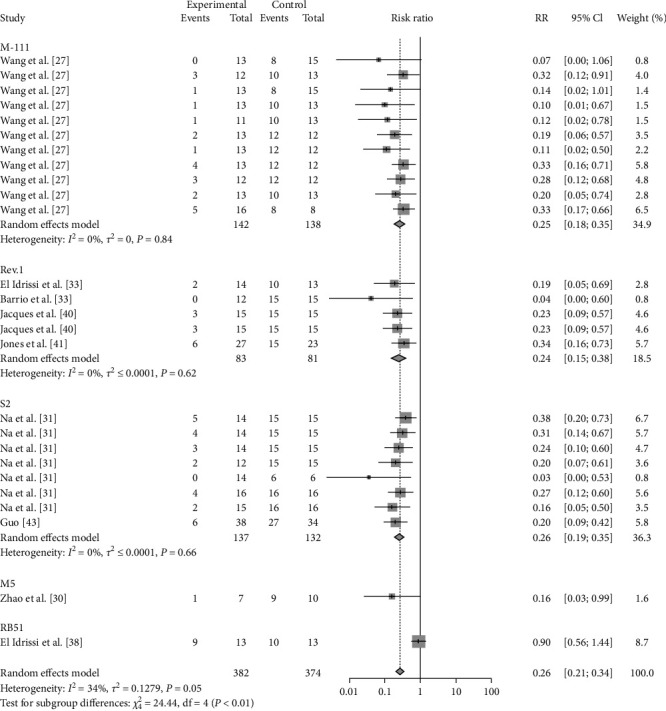
Forest plot of the protective effect of different vaccines against brucellosis infection in females after exposure to virulent *Brucella*.

**Figure 17:**
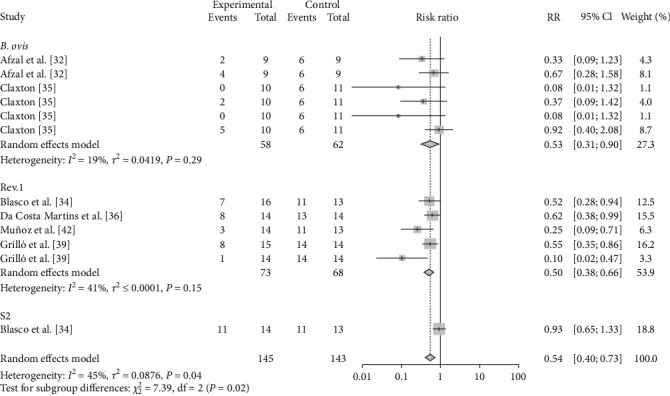
Forest plot of the protective effect of different vaccines against brucellosis infection in males after exposure to virulent *Brucella*.

**Figure 18:**
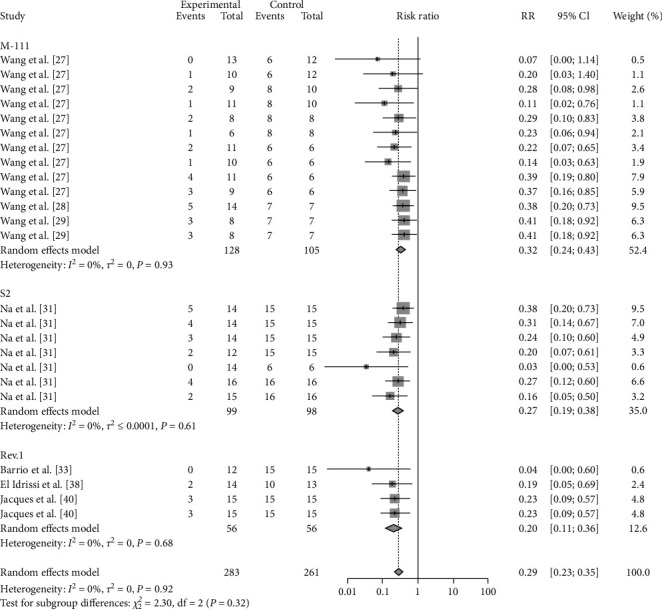
Forest plot of the protective effect of different vaccines against brucellosis infection in pregnant sheep after exposure to virulent *Brucella*.

The effectiveness of vaccination is affected not only by the vaccine itself but also by the age, sex, and health of the immunized animals; for this reason, this paper analyses the above factors. The results showed that females (RR = 0.2639, 95% CI: 0.2059–0.3384; VE = 74%) were more protected (*P*=0.0008) after vaccination than were males (RR = 0.5413, 95% CI: 0.4026–0.7277; VE = 46%) (Table 2, Figure 15, and *Supplementary figure 3*). Compared with those vaccinated with other vaccines (M-111, Rev.1, S2, M5), female sheep vaccinated with the M5 vaccine (RR = 0.1587, 95% CI: 0.0256–0.9858; VE = 84%) exhibited greater protection (*P*  < 0.0001) (Table 2, Figure 16, and *Supplementary figure 3*). Male sheep vaccinated with Rev.1 (RR = 0.5049, 95% CI: 0.3844–0.6633; VE = 50%) were more protected than those vaccinated with the other vaccines (*B. ovis*, S2) (Table 2, Figure 17, and *Supplementary figure 3*). Pregnant ewes, as an important target for protection, showed that vaccination with Rev.1 (RR = 0.2016, 95% CI: 0.1139–0.3569; VE = 80%) was more protective than other vaccines (Table 2, Figure 18, and *Supplementary figure 3*).

**Figure 19:**
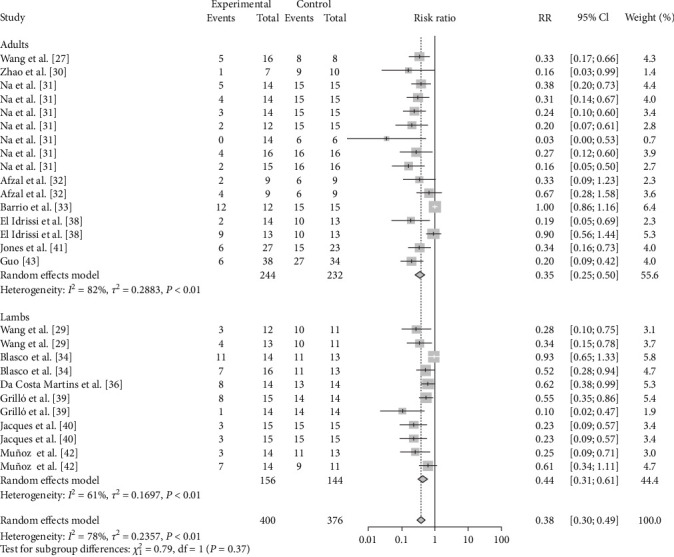
Forest plot of the protective effect of vaccination of sheep of different ages against brucellosis infection after exposure to virulent *Brucella*.

**Figure 20:**
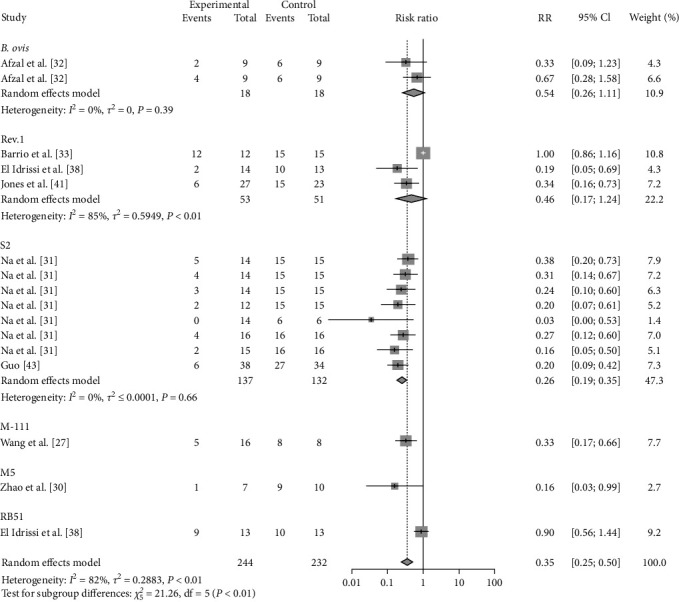
Forest plot of the protective effect of different vaccines against brucellosis infection in adult sheep after exposure to virulent *Brucella*.

**Figure 21:**
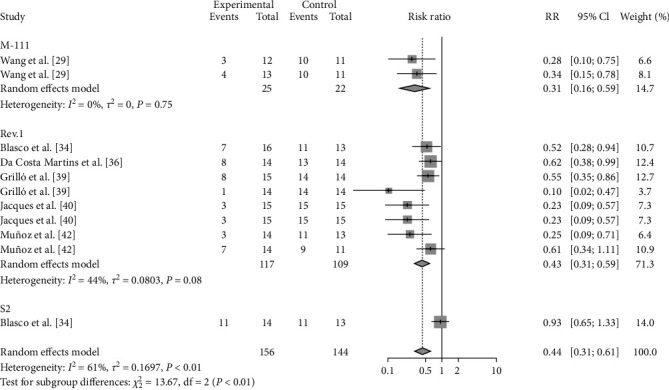
Forest plot of the protective effect of different vaccines against brucellosis infection in lambs after exposure to virulent *Brucella*.

Adult animals (RR = 0.3499, 95% CI: 0.2469–0.4958; VE = 65%) were more protected than lambs (RR = 0.4352, 95% CI: 0.3128–0.6055; VE = 56%) after vaccination (Table 2, Figure 19, and *Supplementary figure 3*). Adult sheep inoculated with M5 (RR = 0.1587, 95% CI: 0.0256–0.9858; VE = 84%) and S2 (RR = 0.2558, 95% CI: 0.1873–0.3493; VE = 74%) were better protected (Table 2, Figure 20, and *Supplementary figure 3*). Lambs inoculated with M-111 (RR = 0.3107, 95% CI: 0.1636–0.5897; VE = 69%) and Rev.1 (RR = 0.4258, 95% CI: 0.3087–0.5873; VE = 57%) were well protected (Table 2, Figure 21, and *Supplementary figure 3*).

### 3.5. Abortion

**Figure 22:**
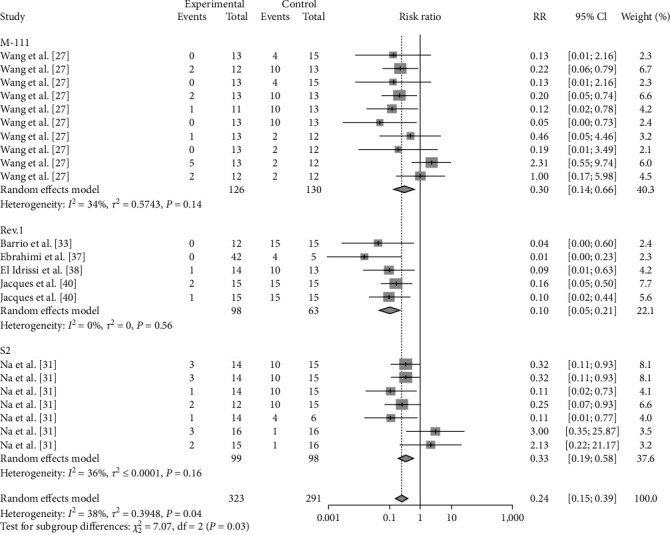
Forest plot of the protective effect of different vaccinations against clinical signs of brucellosis (miscarriage) following exposure to virulent *Brucella*.

As abortion is a major feature of brucellosis, this paper analyses the phenomenon of abortion in vaccinated attacking females. The results showed that Rev.1 (RR = 0.0978, 95% CI: 0.0459–0.2085) vaccination significantly reduced the incidence of abortion in infected females (*P*=0.0299) (Table 5, Figure 22, and *Supplementary figure 3*).

## 4. Discussion

Brucellosis, a globally distributed zoonotic bacterial disease, can infect a wide range of mammals [44]. Sheep, the main type of brucellosis-infected animal, are infected, and infection triggers reduced fertility, the loss of young people, and reduced milk production, leading to significant economic losses in the livestock industry [45]. Vaccines are used as a primary means of preventing brucellosis in sheep, and this study recalculates the efficacy of these vaccines and analyses the relevant influencing factors through a systematic review and meta-analysis, thus providing important information for the development of sheep vaccination protocols.

Based on the susceptible–exposed–infected–recovered–susceptible model of brucellosis, it was observed that it took 16.8 years to eliminate brucellosis from farms after the sheep were vaccinated [46] because the complex and insidious dynamics of brucellosis make it difficult to eradicate brucellosis in most cases, making effective vaccination necessary for the control and eradication of brucellosis in livestock [47]. The results of this study showed that sheep vaccination provided effective protection to the animals (VE = 65%). Efficient animal vaccination is undoubtedly an effective control measure that can significantly reduce the prevalence of brucellosis in livestock, thereby reducing human infections [48]. In addition, we found that female sheep were significantly more effective than male sheep in terms of vaccine immunity after vaccination. First, the majority of the articles included in this study involved pregnant ewes, which, as important breeding and production animals, receive special attention because they have better dietary and feeding conditions and can be better vaccinated with better results [49]. Second, the main animal brucellosis vaccines currently in use are A19, S19, Rev.1, M5, S2, and RB51, of which *Brucella* Rev.1 is one of the most effective [17, 50, 51]. The results of our study showed that both female and male sheep were effectively protected by Rev.1 vaccination, but females were more protected than males, which may explain the significant difference in the protection rates of vaccinated female and male sheep. In our study, it was found that the vaccination of pregnant ewes with Rev.1 produced better protection and reduced the incidence of abortion. Studies have also shown that Rev.1 has good efficacy against brucellosis infections in pregnant ewes, thus reducing economic losses for farmers [52]. Reducing the occurrence of brucellosis-induced abortions in sheep reduces the risk of exposure to infectious vectors and protects the health and safety of the workers involved.

In our study, vaccination had a better effect on adult sheep, although there was no difference in age. Compared with lambs, adult sheep have better body functions, and when the vaccine enters the body, it stimulates an immune response similar to that of a natural infection, inducing a large number of favorable cytokines and generating a sufficient cell-mediated immune response [53]. In the present study, it was found that adult sheep vaccinated with the M5 vaccine had better efficacy than those vaccinated with the S2 vaccine, while lambs vaccinated with M-111 had better vaccination efficacy than those vaccinated with Rev.1. Relevant studies have shown that M5 macrophages have a faster rate of immune antibody production, longer antibody duration, and better levels of postimmunization antibody production than S2 macrophages [54]. In small ruminants, the first step in controlling brucellosis is to vaccinate young animals, which provides lifelong immunity to sheep and thus prevents brucellosis [55]. Since the M-111 vaccine is rarely used in practice, we recommend targeting adult sheep with M5 and lambs with Rev.1, which provides better protection against brucellosis at different ages and thus stimulates a long-lasting protective response in the organism.

The vaccination dose can affect vaccination effectiveness, and we observed that the vaccination effectiveness of Rev.1, S2, and *B. ovis* increased with increasing vaccination dose because, for any single vaccine, larger doses induce better protective immunity [56]. However, we found that high doses of the M-111 vaccine reduced the effectiveness of vaccination. For different vaccines, there are different dosage values; when a certain threshold is exceeded, it can cause poor results. For example, Ebrahimi et al. [37] used different dosage groups to connect the ewes, and after the challenge, the 7.5 × 10^6^ vaccination group triggered a significantly greater proportion of abortions than did the 5 × 10^5^ vaccination group. Therefore, vaccination should be carried out after determining the optimal dose of vaccine required prior to vaccination. Once the vaccination measurements have been determined, different routes of inoculation can also lead to different results. In this study, we found that there was no significant difference in vaccination efficacy between the M-111 and Rev.1 vaccines after using different vaccination methods, while the S2 and *B. ovis* vaccines were more suitable for intramuscular delivery because the intramuscular route is not only the most basic form of inoculation but also generates a stronger and longer lasting antibody response than other forms of inoculation [57]. In summary, we suggest that the optimal vaccination dose should be determined before the inoculation of sheep and that intramuscular injection should be adopted for inoculation to achieve better results and lasting protection of sheep from brucellosis infection.

Limitations of this meta-analysis include, first, the relatively small number of studies and the possibility of publication bias. This paper lacks a comparison between M5 and other vaccines in terms of abortion rate but still reflects to some extent the effect of different vaccines on the abortion rate in sheep. Second, our analyses were limited to published data, and due to the variable nature of the data, it was not possible to further analyze other relevant data; for example, adverse reactions following vaccination were not analyzed in this paper. Finally, this paper only analyses single-dose vaccines and lacks analyses of booster vaccinations.

## 5. Conclusions

Our study highlights the protection offered by different vaccines against brucellosis in sheep. Rev.1 Vaccines protect against brucellosis infection and reduce abortion rates. Sheep should be vaccinated in adulthood, with an emphasis on ewes, and should be vaccinated by intramuscular injection or an inoculation dose >2 × 10^9^ CFU for optimal inoculation results. The results of this systematic evaluation and meta-analysis are useful for considering recommendations for the use of brucellosis vaccines in sheep of different ages and sexes.

## Figures and Tables

**Table 1:** Vaccination and challenge data from trials selected for this systematic review on the efficacy of sheep brucellosis vaccines.

				Vaccination				Challenge	
Reference ID	N Vac	N C	Sex	Strain	Age	Dose	Route	Strain	Dose
Wang et al. [27]	13	15	Female	M-111	N	2.5 × 10^10^	SC	M28	1.59 × 10^9^
	12	13	Female	M-111	N	2.5 × 10^10^	SC	M28	6.3 × 10^9^
	13	15	Female	M-111	N	2.5 × 10^10^	SC	M28	1.59 × 10^9^
	13	12	Female	M-111	N	1.33 × 10^10^	SC	M28	2.475 × 10^9^
	13	12 13	Female Female	M-111 M-111	N N	1.33 × 10^10^ 2.5 × 10^10^	SC SC	M28 M28	4.095 × 10^7^ 2.36 × 10^8^
	11	13	Female	M-111	N	5 × 10^10^	SC	M28	2.36 × 10^8^
	13	15	Female	M-111	N	5 × 10^10^	OR	M28	1.59 × 10^9^
	13	12	Female	M-111	N	2.5 × 10^10^	OR	M28	2.475 × 10^9^
	12	12	Female	M-111	N	2.5 × 10^10^	OR	M28	4.095 × 10^7^

Wang et al. [28]	16	13	Female	M-111	Adults	2.5 × 10^10^	SC	M28	4.215 × 10^8^

Wang et al. [29]	12 13	11 11	N N	M-111 S2	Lambs Lambs	2.5 × 10^10^ 1 × 10^10^	SC OR	M28 M28	1.11 × 10^8^ 1.11 × 10^8^

Zhao et al. [30]	7	10	Female	M5	Adults	0.5 × 10^10^	AI	16 M	1 × 10^7^

Na et al. [31]	14	15	Female	S2	Adults	2.5 × 10^10^	OR	M28	8.725 × 10^7^
	14	15	Female	S2	Adults	5 × 10^10^	OR	M28	8.725 × 10^7^
	14	15	Female	S2	Adults	1.25 × 10^10^	IM	M28	8.725 × 10^7^
	12	15	Female	S2	Adults	0.5 × 10^10^	IM	M28	8.725 × 10^7^
	14	6	Female	S2	Adults	2.5 × 10^10^	OR	M28	5 × 10^8^
	16	16	Female	S2	Adults	1 × 10^10^	OR	M28	1.04 × 10^9^
	15	16	Female	S2	Adults	5 × 10^10^	OR	M28	1.04 × 10^9^

Afzal et al. [32]	9	9	Male	*B. ovis*	Adults	2 × 10^9^	SC	*B. ovis*	5 × 10^9^
	9	9	Male	*B. ovis*	Adults	2 × 10^9^	SC	*B. ovis*	5 × 10^9^

Barrio et al. [33]	12	15	Female	Rev.1	Adults	1 × 10^9^	SC	H38	1.5 × 10^9^

Blasco et al. [34]	14 16	13 13	Male Male	S2 Rev.1	Lambs Lambs	2 × 10^9^ 1.6 × 10^9^	IC IC	*B. ovis* *B. ovis*	6 × 10^9^ 6 × 10^9^

Claxton [35]	10	11	Male	*B. ovis*	N	2.5 × 10^9^	SC	*B. ovis*	2.5 × 10^9^
	10 10	11 11	Male Male	*B. ovis* *B. ovis*	N N	2.5 × 10^9^ 2.5 × 10^9^	SC SC	*B. ovis* B. ovis	2.5 × 10^9^ 2.5 × 10^9^
	10	11	Male	*B. ovis*	N	2.5 × 10^9^	SC	*B. ovis*	2.5 × 10^9^

Da Costa Martins et al. [36]	14	14	Male	Rev.1	Lambs	1.6 × 10^9^	SC	*B. ovis*	1.16 × 10^9^

Ebrahimi et al. [37]	42	5	Female	Rev.1	Adults	N	SC	16 M	4 × 10^9^

El Idrissi et al. [38]	14 13	13 13	Female Female	Rev.1 RB51	Adults Adults	1.73 × 10^8^ 1.1 × 10^10^	IC SC	H38 H38	5 × 10^7^ 5 × 10^7^

Grilló et al. [39]	15	14	Male	Rev.1	Lambs	1 × 10^9^	SC	*B. ovis*	1.7 × 10^9^
	14	14	Male	Rev.1	Lambs	1.2 × 10^9^	IC	*B. ovis*	1.7 ×10^9^

Jacques et al. [40]	15 15	15 15	Female Female	Rev.1 Rev.1	Adults Adults	1.1 × 10^10^ 1.3 × 10^9^	SC IC	H38 H38	5.1 × 10^7^ 5.1 × 10^7^

Jones et al. [41]	27	23	Female	Rev.1	Adults	2.22 × 10^9^	SC	*B. melitensis*	6 × 10^9^

Muñoz et al. [42]	14 14	13 13	Male Male	Rev.1 Rev.1	Lambs Lambs	2.5 × 10^10^ 2 × 10^9^	SC SC	*B. ovis* *B. ovis*	2.5 × 10^9^ 2.5 × 10^9^

Guo [43]	38	34	Female	S2	Adults	1 × 10^10^	OR	M28	N

N Vac, Vaccine group; N C, Vontrol group; N, None.

**Table 2:** Postchallenge results in brucellosis-vaccinated and unvaccinated animals.

	Number of studies	RR (95% CI^*∗*^)	Heterogeneity		Univariate meta-regression		
			*χ*²	*P*-value	*I*² (%)	*P*-value	Coefficient (95% CI)
Vaccine						0.0401	−0.6942 (−1.4645 to −0.0339)
M-111	12	0.2527 [0.1839; 0.3473]	5.75	0.89	0.0		
Rev.1	11	0.3825 [0.2792; 0.5242]	17.98	0.06	44.4
S2	10	0.3490 [0.2334; 0.5220]	29.24	<0.01	69.2
* B. ovis*	6	0.5298 [0.3126; 0.8979]	6.16	0.29	18.8
M5	1	0.1587 [0.0256; 0.9858]	0.00	—	—
RB51	1	0.9000 [0.5631; 1.4386]	0.00	—	—
Gender						0.0008	0.6875 (0.2851–1.0899)
Female	26	0.2639 [0.2059; 0.3384]	37.80	0.05	33.9		
Male	12	0.5413 [0.4026; 0.7277]	20.16	0.04	45.4
Female						<0.0001	1.2797 (0.7031–1.8562)
M-111	11	0.2503 [0.1790; 0.3500]	5.72	0.84	0.0		
Rev.1	5	0.2432 [0.1538; 0.3844]	2.65	0.62	0.0
S2	8	0.2558 [0.1873; 0.3493]	4.99	0.66	—
M5	1	0.1587 [0.0256; 0.9858]	0.00	—	—
RB51	1	0.9000 [0.5631; 1.4386]	0.00	—	—
Male						0.7618	−0.0863 (−0.6445 to 0.4718)
* B. ovis*	6	0.5298 [0.3126; 0.8979]	6.16	0.29	18.8		
Rev.1	5	0.5049 [0.3844; 0.6633]	6.73	0.15	40.5
S2	1	0.9286 [0.6488; 1.3290]	0.00	—	—
Age						0.5052	0.1692 (−0.3285 to 0.6670)
Adult	16	0.3499 [0.2469; 0.4958]	84.46	<0.01	82.2		
Lamb	11	0.4352 [0.3128; 0.6055]	25.73	<0.01	61.1
Adult						0.1405	−0.7556 (−1.7604 to 0.2492)
* B. ovis*	2	0.5399 [0.2626; 1.1101]	0.75	0.39	0.0		
Rev.1	3	0.4630 [0.1729; 1.2398]	13.25	<0.01	84.9
S2	8	0.2558 [0.1873; 0.3493]	4.99	0.66	0.0
M-111	1	0.3333 [0.1685; 0.6595]	0.00	—	—
M5	1	0.1587 [0.0256; 0.9858]	0.00	—	—
RB51	1	0.9000 [0.5631; 1.4386]	0.00	—	—
Lamb						0.3746	0.3484 (−0.4206 to 1.1174)
M-111	2	0.3107 [0.1636; 0.5897]	0.10	0.75	0.0		
Rev.1	8	0.4258 [0.3087; 0.5873]	12.60	0.08	44.5
S2	1	0.9286 [0.6488; 1.3290]	0.00	—	—
Pregnant						0.1454	−0.4724 (−1.1083 to 0.1635)
M-111	13	0.3234 [0.2445; 0.4277]	5.82	0.93	0.0		
Rev.1	4	0.2016 [0.1139; 0.3569]	1.49	0.68	0.0
S2	7	0.2694 [0.1913; 0.3793]	4.47	0.61	0.0

CI*⁣*^*∗*^: confidence interval.

**Table 3:** Postchallenge results in animals vaccinated with different doses of the brucellosis vaccine and unvaccinated animals.

Vaccine	Number of studies	RR (95% CI^*∗*^)	Heterogeneity		Univariate metaregression		
			*χ*²	*P*-value	*I* ^2^ (%)	*P*-value	Coefficient (95% CI)
M-111						0.2663	0.5552 (−0.4237 to 1.5341)
<2.5 × 10^10^	2	0.1545 [0.0629; 0.3795]	0.28	0.59	0.0		
≥2.5 × 10^10^	10	0.2692 [0.1826; 0.3967]	8.41	0.49	0.0
Rev.1						0.2877	0.3865 (−0.3259 to 1.0989)
≤2 × 10^9^	7	0.3604 [0.2208; 0.5882]	13.29	0.04	54.8		
>2 × 10^9^	3	0.2790 [0.1673; 0.4653]	0.50	0.78	0.0
S2						0.6053	0.2300 (−0.6422 to 1.1021)
≤1 × 10^10^	5	0.3260 [0.1671; 0.6358]	24.44	<0.01	83.6		
>1 × 10^10^	5	0.2999 [0.2016; 0.4461]	4.15	0.39	3.5
*B. ovis*						0.8178	0.1657 (−1.2439 to 1.5753)
2 × 10^9^	2	0.5399 [0.2626; 1.1101]	0.75	0.39	0.0		
2.5 × 10^9^	4	0.3736 [0.1216; 1.1482]	5.40	0.14	44.4

CI*⁣*^*∗*^: confidence interval.

**Table 4:** Postchallenge results of the brucellosis vaccine in different modes of vaccination and unvaccinated animals.

Vaccine	Number of studies	RR (95% CI^*∗*^)	Heterogeneity		Univariate metaregression		
			*χ* ^2^	*P*-value	*I* ^2^ (%)	*P*-value	Coefficient (95% CI)
M-111						0.5364	−0.2129 (−0.8877 to 0.4620)
Oral gavage	3	0.2913 [0.1678; 0.5057]	0.63	0.73	0.0		
Subcutaneous	9	0.2355 [0.1596; 0.3474]	4.73	0.79	0.0
Rev.1						0.4043	0.3287 (−0.4439 to 1.1013)
Intraconjunctival	4	0.2653 [0.1313; 0.5361]	5.77	0.04	48.0		
Subcutaneous	6	0.3993 [0.2654; 0.6008]	9.48	0.78	47.2
S2						<0.0001	−1.2447 (−1.2447 to −0.7719)
Intramuscular	1	0.2414 [0.0969; 0.6012]	0.00	—	—		
Intraconjunctival	1	0.9286 [0.6488; 1.3290]	0.00	—	—
Oral gavage	8	0.2675 [0.1965; 0.3640]	5.53	0.62	0.0

CI*⁣*^*∗*^: confidence interval.

**Table 5:** Post-challenge abortion outcomes in brucellosis-vaccinated and unvaccinated animals.

Vaccine	Number of studies	RR (95% CI^*∗*^)		Heterogeneity	Univariate metaregression		
			*χ* ^2^	*P*-value	*I* ^2^ (%)	*P*-value	Coefficient (95% CI)
						0.0299	−1.2644 (−2.4057 to −0.1231)

M-111	10	0.3015 [0.1378; 0.6597]	13.60	0.14	33.8		
Rev.1	5	0.0978 [0.0459; 0.2085]	2.99	0.56	0.0
S2	7	0.3329 [0.1916; 0.5786]	9.31	0.16	35.6

CI*⁣*^*∗*^: confidence interval.

## Data Availability

The datasets used in this work are accessible upon reasonable request from the corresponding author.

## Abbreviations

PRISMA: Preferred reporting items for systematic reviews and meta-analyses
GRADE: Grading of recommendations assessment, development, and evaluation.

## Additional Points

List of publications for the author (Lian-Min Li): Li LM, Wang Q, Shi JF, Li T, Zhao B, Ma QX, Liu HY, Su N, Cai RP, Zeng FL, Gong QL, Shi K, Li JM, Liu F, Du R. Seroprevalence and potential risk factors of brucellosis in sheep from America, Africa and Asia regions: A systematic review and meta-analysis. Res Vet Sci. 2023 Dec; 165: 105048. doi: 10.1016/j.rvsc.2023.105048.

## Supplementary Materials

Supplementary 1 Appendix 1: strategy for searching PubMed, Cochrane Library, Science Direct, VIP, Wan Fang, and CNKI.

Supplementary 2 Table 1: PRISMA Checklist item. Table 2: Egger's test for publication bias.

Supplementary 3 Figure 1: funnel chart of the protective effect of vaccination with different species against brucellosis infection after exposure to virulent *Brucella*. Figure 2: funnel chart of the protective effect of different doses of M-111 against brucellosis infection after exposure to virulent *Brucella*. Figure 3: funnel chart of the protective effect of different doses of Rev.1 inoculation against brucellosis infection after exposure to virulent *Brucella*. Figure 4: funnel chart of the protective effect of different doses of S2 inoculation against brucellosis infection after exposure to virulent *Brucella*. Figure 5: funnel chart of the protective effect of different doses of *B. ovis* inoculation against brucellosis infection after exposure to virulent *Brucella*. Figure 6. funnel chart of the protective effect of different inoculation modes of M-111 against brucellosis infection after exposure to virulent *Brucella*. Figure 7. funnel chart of the protective effect of different inoculation modes of Rev.1 against brucellosis infection after exposure to virulent *Brucella*. Figure 8: funnel chart of the protective effect of different inoculation modes of S2 against brucellosis infection after exposure to virulent *Brucella*. Figure 9: funnel chart of the protective effect of vaccination of different sexes against brucellosis infection after exposure to virulent *Brucella*. Figure 10: funnel chart of the protective effect of different vaccines against brucellosis infection in females after exposure to virulent *Brucella*. Figure 11: funnel chart of the protective effect of different vaccines against brucellosis infection in males after exposure to virulent *Brucella*. Figure 12: funnel chart of the protective effect of different vaccines against brucellosis infection in pregnant sheep after exposure to virulent *Brucella*. Figure 13: funnel chart of the protective effect of vaccination of sheep of different ages against brucellosis infection after exposure to virulent *Brucella*. Figure 14: funnel chart of the protective effect of different vaccines against brucellosis infection in adult sheep after exposure to virulent *Brucella*. Figure 15: funnel chart of the protective effect of different vaccines against brucellosis infection in lambs after exposure to virulent *Brucella*. Figure 16: funnel chart of the protective effect of different vaccinations against clinical signs of brucellosis (miscarriage) following exposure to virulent *Brucella*.
