# Supplementary material for: Efficacy of Brucella Vaccines in Sheep: A Systematic Review and Meta-Analysis
Source: Transbound Emerg Dis. 2024 Jul 26;2024:5524768. doi: 10.1155/2024/5524768 (PMC12016899; doi:10.1155/2024/5524768)
Supplement: Supplementary 1 — Appendix 1: strategy for searching PubMed, Cochrane Library, Science Direct, VIP, Wan Fang, and CNKI. [file 5524768.f1.docx]

**S1 Appendix.**

Strategy for searching PubMed

("Sheep"[MeSH Terms] OR ("Sheep"[MeSH Terms] OR "Sheep"[All Fields] OR "ovis"[All Fields]) OR ("Sheep"[MeSH Terms] OR "Sheep"[All Fields] OR ("dall"[All Fields] AND "Sheep"[All Fields]) OR "dall sheep"[All Fields]) OR ("Sheep"[MeSH Terms] OR "Sheep"[All Fields] OR ("ovis"[All Fields] AND "dalli"[All Fields]) OR "ovis dalli"[All Fields]) OR ("Sheep"[MeSH Terms] OR "Sheep"[All Fields] OR ("Sheep"[All Fields] AND "dall"[All Fields]) OR "sheep dall"[All Fields])) AND ("Vaccines"[MeSH Terms] OR ("vaccin"[Supplementary Concept] OR "vaccin"[All Fields] OR "Vaccination"[MeSH Terms] OR "Vaccination"[All Fields] OR "vaccinable"[All Fields] OR "vaccinal"[All Fields] OR "vaccinate"[All Fields] OR "vaccinated"[All Fields] OR "vaccinates"[All Fields] OR "vaccinating"[All Fields] OR "vaccinations"[All Fields] OR "vaccination s"[All Fields] OR "vaccinator"[All Fields] OR "vaccinators"[All Fields] OR "vaccine s"[All Fields] OR "vaccined"[All Fields] OR "Vaccines"[MeSH Terms] OR "Vaccines"[All Fields] OR "vaccine"[All Fields] OR "vaccins"[All Fields]) OR ("Vaccination"[MeSH Terms] OR ("vaccin"[Supplementary Concept] OR "vaccin"[All Fields] OR "Vaccination"[MeSH Terms] OR "Vaccination"[All Fields] OR "vaccinable"[All Fields] OR "vaccinal"[All Fields] OR "vaccinate"[All Fields] OR "vaccinated"[All Fields] OR "vaccinates"[All Fields] OR "vaccinating"[All Fields] OR "vaccinations"[All Fields] OR "vaccination s"[All Fields] OR "vaccinator"[All Fields] OR "vaccinators"[All Fields] OR "vaccine s"[All Fields] OR "vaccined"[All Fields] OR "Vaccines"[MeSH Terms] OR "Vaccines"[All Fields] OR "vaccine"[All Fields] OR "vaccins"[All Fields]) OR ("Vaccination"[MeSH Terms] OR "Vaccination"[All Fields] OR ("Immunization"[All Fields] AND "active"[All Fields]) OR "immunization active"[All Fields]) OR ("active immunisation"[All Fields] OR "Vaccination"[MeSH Terms] OR "Vaccination"[All Fields] OR ("active"[All Fields] AND "Immunization"[All Fields]) OR "active immunization"[All Fields]) OR ("active immunisations"[All Fields] OR "Vaccination"[MeSH Terms] OR "Vaccination"[All Fields] OR ("active"[All Fields] AND "immunizations"[All Fields]) OR "active immunizations"[All Fields]) OR ("Vaccination"[MeSH Terms] OR "Vaccination"[All Fields] OR ("immunizations"[All Fields] AND "active"[All Fields]) OR "immunizations active"[All Fields]))) AND ("Brucellosis"[MeSH Terms] OR ("Brucellosis"[MeSH Terms] OR "Brucellosis"[All Fields] OR "brucelloses"[All Fields]) OR ("Brucellosis"[MeSH Terms] OR "Brucellosis"[All Fields] OR ("malta"[All Fields] AND "fever"[All Fields]) OR "malta fever"[All Fields]) OR ("Brucellosis"[MeSH Terms] OR "Brucellosis"[All Fields] OR ("fever"[All Fields] AND "malta"[All Fields]) OR "fever malta"[All Fields]) OR ("Brucellosis"[MeSH Terms] OR "Brucellosis"[All Fields] OR ("gibraltar"[All Fields] AND "fever"[All Fields]) OR "gibraltar fever"[All Fields]) OR ("Brucellosis"[MeSH Terms] OR "Brucellosis"[All Fields] OR ("fever"[All Fields] AND "gibraltar"[All Fields]) OR "fever gibraltar"[All Fields]) OR ("Brucellosis"[MeSH Terms] OR "Brucellosis"[All Fields] OR ("rock"[All Fields] AND "fever"[All Fields]) OR "rock fever"[All Fields]) OR ("Brucellosis"[MeSH Terms] OR "Brucellosis"[All Fields] OR ("fever"[All Fields] AND "rock"[All Fields])) OR ("Brucellosis"[MeSH Terms] OR "Brucellosis"[All Fields] OR ("cyprus"[All Fields] AND "fever"[All Fields]) OR "cyprus fever"[All Fields]) OR ("Brucellosis"[MeSH Terms] OR "Brucellosis"[All Fields] OR ("fever"[All Fields] AND "cyprus"[All Fields]) OR "fever cyprus"[All Fields]) OR ("Brucellosis"[MeSH Terms] OR "Brucellosis"[All Fields] OR ("brucella"[All Fields] AND "infection"[All Fields]) OR "brucella infection"[All Fields]) OR ("Brucellosis"[MeSH Terms] OR "Brucellosis"[All Fields] OR ("brucella"[All Fields] AND "infections"[All Fields]) OR "brucella infections"[All Fields]) OR ("Brucellosis"[MeSH Terms] OR "Brucellosis"[All Fields] OR ("infection"[All Fields] AND "brucella"[All Fields]) OR "infection brucella"[All Fields]) OR ("Brucellosis"[MeSH Terms] OR "Brucellosis"[All Fields] OR ("undulant"[All Fields] AND "fever"[All Fields]) OR "undulant fever"[All Fields]) OR ("Brucellosis"[MeSH Terms] OR "Brucellosis"[All Fields] OR ("fever"[All Fields] AND "undulant"[All Fields]) OR "fever undulant"[All Fields]) OR ("Brucellosis"[MeSH Terms] OR "Brucellosis"[All Fields] OR ("Brucellosis"[All Fields] AND "pulmonary"[All Fields]) OR "brucellosis pulmonary"[All Fields]) OR ("Brucellosis"[MeSH Terms] OR "Brucellosis"[All Fields] OR ("brucelloses"[All Fields] AND "pulmonary"[All Fields])) OR ("Brucellosis"[MeSH Terms] OR "Brucellosis"[All Fields] OR ("pulmonary"[All Fields] AND "brucelloses"[All Fields])) OR ("Brucellosis"[MeSH Terms] OR "Brucellosis"[All Fields] OR ("pulmonary"[All Fields] AND "Brucellosis"[All Fields]) OR "pulmonary brucellosis"[All Fields])) AND ("Immunization"[MeSH Terms] OR ("immune"[All Fields] OR "immuned"[All Fields] OR "immunes"[All Fields] OR "immunisation"[All Fields] OR "Vaccination"[MeSH Terms] OR "Vaccination"[All Fields] OR "Immunization"[All Fields] OR "Immunization"[MeSH Terms] OR "immunisations"[All Fields] OR "immunizations"[All Fields] OR "immunise"[All Fields] OR "immunised"[All Fields] OR "immuniser"[All Fields] OR "immunisers"[All Fields] OR "immunising"[All Fields] OR "immunities"[All Fields] OR "immunity"[MeSH Terms] OR "immunity"[All Fields] OR "immunization s"[All Fields] OR "immunize"[All Fields] OR "immunized"[All Fields] OR "immunizer"[All Fields] OR "immunizers"[All Fields] OR "immunizes"[All Fields] OR "immunizing"[All Fields]) OR ("Immunization"[MeSH Terms] OR "Immunization"[All Fields] OR ("sensitization"[All Fields] AND "immunologic"[All Fields]) OR "sensitization immunologic"[All Fields]) OR ("Immunization"[MeSH Terms] OR "Immunization"[All Fields] OR ("sensitization"[All Fields] AND "immunological"[All Fields]) OR "sensitization immunological"[All Fields]) OR ("immunological sensitisation"[All Fields] OR "Immunization"[MeSH Terms] OR "Immunization"[All Fields] OR ("immunological"[All Fields] AND "sensitization"[All Fields]) OR "immunological sensitization"[All Fields]) OR ("Immunization"[MeSH Terms] OR "Immunization"[All Fields] OR ("immunological"[All Fields] AND "sensitizations"[All Fields])) OR ("Immunization"[MeSH Terms] OR "Immunization"[All Fields] OR ("sensitizations"[All Fields] AND "immunological"[All Fields])) OR ("Immunization"[MeSH Terms] OR "Immunization"[All Fields] OR ("immunologic"[All Fields] AND "stimulation"[All Fields]) OR "immunologic stimulation"[All Fields]) OR ("adjuvants immunologic"[Pharmacological Action] OR "adjuvants, immunologic"[MeSH Terms] OR ("adjuvants"[All Fields] AND "immunologic"[All Fields]) OR "immunologic adjuvants"[All Fields] OR "immunostimulant"[All Fields] OR "immunostimulants"[All Fields] OR "Immunization"[MeSH Terms] OR "Immunization"[All Fields] OR "immunostimulation"[All Fields] OR "immunostimulating"[All Fields] OR "immunostimulations"[All Fields] OR "immunostimulative"[All Fields] OR "immunostimulator"[All Fields] OR "immunostimulators"[All Fields]) OR ("Immunization"[MeSH Terms] OR "Immunization"[All Fields] OR ("immunological"[All Fields] AND "stimulation"[All Fields]) OR "immunological stimulation"[All Fields]) OR ("Immunization"[MeSH Terms] OR "Immunization"[All Fields] OR ("immunological"[All Fields] AND "stimulations"[All Fields]) OR "immunological stimulations"[All Fields]) OR ("Immunization"[MeSH Terms] OR "Immunization"[All Fields] OR ("stimulation"[All Fields] AND "immunological"[All Fields]) OR "stimulation immunological"[All Fields]) OR ("Immunization"[MeSH Terms] OR "Immunization"[All Fields] OR ("stimulations"[All Fields] AND "immunological"[All Fields])) OR ("Immunization"[MeSH Terms] OR "Immunization"[All Fields] OR ("immunologic"[All Fields] AND "sensitization"[All Fields]) OR "immunologic sensitization"[All Fields]) OR ("Immunization"[MeSH Terms] OR "Immunization"[All Fields] OR ("stimulation"[All Fields] AND "immunologic"[All Fields]) OR "stimulation immunologic"[All Fields]) OR ("Immunization"[MeSH Terms] OR "Immunization"[All Fields] OR "variolation"[All Fields]) OR ("Immunization"[MeSH Terms] OR "Immunization"[All Fields] OR "variolations"[All Fields]))

Strategy for searching Cochrane1

#1 MeSH descriptor: [Sheep] explode all trees

#2 MeSH descriptor: [Vaccines] explode all trees

#3 MeSH descriptor: [Brucellosis] explode all trees 7

#4 #2

#5 #1 and #4 and #3

Strategy for searching Science Direct, VIP, Wan Fang, and CNKI

Search for (Sheep and *Brucella* or Brucellosis) and (Vaccines and Immunizations) by keyword, unlimited years
