# Supplementary material for: Efficacy of Brucella Vaccines in Sheep: A Systematic Review and Meta-Analysis
Source: Transbound Emerg Dis. 2024 Jul 26;2024:5524768. doi: 10.1155/2024/5524768 (PMC12016899; doi:10.1155/2024/5524768)
Supplement: Supplementary 2 — Table 1: PRISMA Checklist item. Table 2: Egger's test for publication bias. [file 5524768.f2.docx]

**Table S1.** PRISMA Checklist item.

| **Section/topic** | **#** | **Checklist item** | **Reported on page #** |
| --- | --- | --- | --- |
| **TITLE** |  |  |  |
| Title | 1 | Efficacy of Brucella vaccines in sheep: a systematic review and meta-analysis | 1 |
| **ABSTRACT** |  |  |  |
| Structured summary | 2 | Provide a structured summary including, as applicable: background; objectives; data sources; study eligibility criteria, participants, and interventions; study appraisal and synthesis methods; results; limitations; conclusions and implications of key findings; systematic review registration number. | 2 |
| **INTRODUCTION** |  |  |  |
| Rationale | 3 | Describe the rationale for the review in the context of what is already known. | 4-6 |
| Objectives | 4 | Provide an explicit statement of questions being addressed with reference to participants, interventions, comparisons, outcomes, and study design (PICOS). | 4-6 |
| **METHODS** |  |  |  |
| Protocol and registration | 5 | Indicate if a review protocol exists, if and where it can be accessed (e.g., Web address), and, if available, provide registration information including registration number. | 4-6 |
| Eligibility criteria | 6 | Specify study characteristics (e.g., PICOS, length of follow-up) and report characteristics (e.g., years considered, language, publication status) used as criteria for eligibility, giving rationale. | 4-6 |
| Information sources | 7 | Describe all information sources (e.g., databases with dates of coverage, contact with study authors to identify additional studies) in the search and date last searched. | 4-6 |
| Search | 8 | Present full electronic search strategy for at least one database, including any limits used, such that it could be repeated. | 4-6 |
| Study selection | 9 | State the process for selecting studies (i.e., screening, eligibility, included in systematic review, and, if applicable, included in the meta-analysis). | 4-6 |
| Data collection process | 10 | Describe method of data extraction from reports (e.g., piloted forms, independently, in duplicate) and any processes for obtaining and confirming data from investigators. | 4-5 |
| Data items | 11 | List and define all variables for which data were sought (e.g., PICOS, funding sources) and any assumptions and simplifications made. | 4-5 |
| Risk of bias in individual studies | 12 | Describe methods used for assessing risk of bias of individual studies (including specification of whether this was done at the study or outcome level), and how this information is to be used in any data synthesis. | 5-6 |
| Summary measures | 13 | State the principal summary measures (e.g., risk ratio, difference in means). | 5-6 |
| Synthesis of results | 14 | Describe the methods of handling data and combining results of studies, if done, including measures of consistency (e.g., I^2^) for each meta-analysis. | 5-6 |
| Risk of bias across studies | 15 | Specify any assessment of risk of bias that may affect the cumulative evidence (e.g., publication bias, selective reporting within studies). | 6 |
| Additional analyses | 16 | Describe methods of additional analyses (e.g., sensitivity or subgroup analyses, meta-regression), if done, indicating which were pre-specified. | 6 |
| **RESULTS** |  |  |  |
| Study selection | 17 | Give numbers of studies screened, assessed for eligibility, and included in the review, with reasons for exclusions at each stage, ideally with a flow diagram. | 6, Fig 1 |
| Study characteristics | 18 | For each study, present characteristics for which data were extracted (e.g., study size, PICOS, follow-up period) and provide the citations. | 6, Tab 1 |
| Risk of bias within studies | 19 | Present data on risk of bias of each study and, if available, any outcome level assessment (see item 12). | 7-9, Fig 2-5 |
| Results of individual studies | 20 | For all outcomes considered (benefits or harms), present, for each study: (a) simple summary data for each intervention group (b) effect estimates and confidence intervals, ideally with a forest plot. | 7-9, Tab 2-3 |
| Synthesis of results | 21 | Present results of each meta-analysis done, including confidence intervals and measures of consistency. | 7-9, Tab 2-3 |
| Risk of bias across studies | 22 | Present results of any assessment of risk of bias across studies (see Item 15). | 7-9, Fig 2-5 |
| Additional analysis | 23 | Give results of additional analyses, if done (e.g., sensitivity or subgroup analyses, meta-regression [see Item 16]). | 7-9, Fig 2-5 |
| **DISCUSSION** |  |  |  |
| Summary of evidence | 24 | Summarize the main findings including the strength of evidence for each main outcome; consider their relevance to key groups (e.g., healthcare providers, users, and policy makers). | 9-12 |
| Limitations | 25 | Discuss limitations at study and outcome level (e.g., risk of bias), and at review-level (e.g., incomplete retrieval of identified research, reporting bias). | 11 |
| Conclusions | 26 | Provide a general interpretation of the results in the context of other evidence, and implications for future research. | 12 |
| **FUNDING** |  |  |  |
| Funding | 27 | Describe sources of funding for the systematic review and other support (e.g., supply of data); role of founders for the systematic review. | 13 |

*From:* Moher D, Liberati A, Tetzlaff J, Altman DG, The PRISMA Group (2009). Preferred Reporting Items for Systematic Reviews and Meta-Analyses: The PRISMA Statement. PLoS Med 6(6): e1000097. doi:10.1371/journal.pmed1000097

For more information, visit: **www.prisma-statement.org**.

**References**

1. **Wang W, Lv XZ, Hao YF et al. The research of Brucella melitensis strain M-111 vaccine for the routes, dose, number of times and duration of sheep [J]. Chinese Journal of Zoonoses,1991(02):63-66.**
2. **Wang W, Wang RL, Fan BY et al.Determination of the duration of immunity of Brucellosis M-111 vaccine in sheep[J].Chinese Journal of Veterinary Drug,1993,27(01):25-27.**
3. **Wang W, Lv XZ, Hao YF et al. Determination of the two-year immunity period of Brucella M-111 vaccine in lambs[J]. Chinese Journal of Zoonoses,1992(04):59-60.**
4. **Zhao YQ, Xie SD, Bai LX et al. Determination of the efficacy of aerosol immunisation with Brucella 5 vaccine for large-scale application - a test of the efficacy of two consecutive immunisations followed by 2.5 years of immunisation duration[J]. Chinese Journal of Veterinary Medicine,1984(01):12-14.**
5. **Na S, Wu ZS, Tang GH.Immunization of sheep, goats, catteand pigs with BR, suis strain 2 vaccine IV. Oral Immunization of sheep and goats[J]. Acta Veterinaria et Zootechnica Sinica,1998(04):248-249.**
6. **Afzal M, Tengerdy RP, Ellis RP, Kimberling CV, Morris CJ. Protection of rams against epididymitis by a Brucella ovis-vitamin E adjuvant vaccine. Vet Immunol Immunopathol. 1984 Oct;7(3-4):293-304. doi: 10.1016/0165-2427(84)90087-4. PMID: 6506451.**
7. **Barrio MB, Grilló MJ, Muñoz PM, Jacques I, González D, de Miguel MJ, Marín CM, Barberán M, Letesson JJ, Gorvel JP, Moriyón I, Blasco JM, Zygmunt MS. Rough mutants defective in core and O-polysaccharide synthesis and export induce antibodies reacting in an indirect ELISA with smooth lipopolysaccharide and are less effective than Rev 1 vaccine against Brucella melitensis infection of sheep. Vaccine. 2009 Mar 10;27(11):1741-9. doi: 10.1016/j.vaccine.2009.01.025. Epub 2009 Jan 30. PMID: 19186196.**
8. **Blasco JM, Marín C, Jiménez de Bagüés MP, Barberán M. Efficacy of Brucella suis strain 2 vaccine against Brucella ovis in rams. Vaccine. 1993 Oct;11(13):1291-4. doi: 10.1016/0264-410x(93)90097-h. PMID: 8296481.**
9. **Claxton PD. Brucella ovis vaccination of rams. A comparison of two commercial vaccines and two methods of vaccination. Aust Vet J. 1968 Feb;44(2):48-54. doi: 10.1111/j.1751-0813.1968.tb04953.x. PMID: 5688907.**
10. **Da Costa Martins R, Irache JM, Blasco JM, Muñoz MP, Marín CM, Jesús Grilló M, Jesús De Miguel M, Barberán M, Gamazo C. Evaluation of particulate acellular vaccines against Brucella ovis infection in rams. Vaccine. 2010 Apr 9;28(17):3038-46. doi: 10.1016/j.vaccine.2009.10.073. Epub 2009 Nov 1. PMID: 19887131.**
11. **Ebrahimi M, Nejad RB, Alamian S, Mokhberalsafa L, Abedini F, Ghaderi R, Jalali HR. Safety and efficacy of reduced doses of Brucella melitensis strain Rev. 1 vaccine in pregnant Iranian fat-tailed ewes. Vet Ital. 2012 Oct-Dec;48(4):405-12. PMID: 23277121.**
12. **el Idrissi AH, Benkirane A, el Maadoudi M, Bouslikhane M, Berrada J, Zerouali A. Comparison of the efficacy of Brucella abortus strain RB51 and Brucella melitensis Rev. 1 live vaccines against experimental infection with Brucella melitensis in pregnant ewes. Rev Sci Tech. 2001 Dec;20(3):741-7. doi: 10.20506/rst.20.3.1305. PMID: 11732416.**
13. **Grilló MJ, Marín CM, Barberán M, de Miguel MJ, Laroucau K, Jacques I, Blasco JM. Efficacy of bp26 and bp26/omp31 B. melitensis Rev.1 deletion mutants against Brucella ovis in rams. Vaccine. 2009 Jan 7;27(2):187-91. doi: 10.1016/j.vaccine.2008.10.065. Epub 2008 Nov 11. PMID: 19007836.**
14. **Jacques I, Verger JM, Laroucau K, Grayon M, Vizcaino N, Peix A, Cortade F, Carreras F, Guilloteau LA. Immunological responses and protective efficacy against Brucella melitensis induced by bp26 and omp31 B. melitensis Rev.1 deletion mutants in sheep. Vaccine. 2007 Jan 15;25(5):794-805. doi: 10.1016/j.vaccine.2006.09.051. Epub 2006 Sep 27. PMID: 17070627.**
15. **JONES LM, ENTESSAR F, ARDALAN A. COMPARISON OF LIVING VACCINES IN PRODUCING IMMUNITY AGAINST NATURAL BRUCELLA MELITENSIS INFECTION IN SHEEP AND GOATS IN IRAN. J Comp Pathol. 1964 Jan;74:17-30. doi: 10.1016/s0368-1742(64)80003-5. PMID: 14105237.**
16. **Muñoz PM, Conde-Álvarez R, Andrés-Barranco S, de Miguel MJ, Zúñiga-Ripa A, Aragón-Aranda B, Salvador-Bescós M, Martínez-Gómez E, Iriarte M, Barberán M, Vizcaíno N, Moriyón I, Blasco JM. A Brucella melitensis H38ΔwbkF rough mutant protects against Brucella ovis in rams. Vet Res. 2022 Mar 2;53(1):16. doi: 10.1186/s13567-022-01034-z. PMID: 35236406; PMCID: PMC8889640.**
17. **Guo LC. Studies on the protective power and duration of oral immunity of Brucella II vaccine in sheep[J]. Chinese Journal of Veterinary Medicine,1983(06):50-51.**

**Table S2.** Egger’s for publication bias.

| bias | se. bias | t | df | *P*-value |
| --- | --- | --- | --- | --- |
| -2.6458 | 0.2599 | -10.18 | 39 | < 0.0001 |
