# Supplementary material for: Efficacy of Brucella Vaccines in Sheep: A Systematic Review and Meta-Analysis
Source: Transbound Emerg Dis. 2024 Jul 26;2024:5524768. doi: 10.1155/2024/5524768 (PMC12016899; doi:10.1155/2024/5524768)
Supplement: Supplementary 3 — Figure 1: funnel chart of the protective effect of vaccination with different species against brucellosis infection after exposure to virulent Brucella. Figure 2: funnel chart of the protective effect of different doses of M-111 against brucellosis infection after exposure to virulent Brucella. Figure 3: funnel chart of the protective effect of different doses of Rev.1 inoculation against brucellosis infection after exposure to virulent Brucella. Figure 4: funnel chart of the protective effect of different doses of S2 inoculation against brucellosis infection after exposure to virulent Brucella. Figure 5: funnel chart of the protective effect of different doses of B. ovis inoculation against brucellosis infection after exposure to virulent Brucella. Figure 6. funnel chart of the protective effect of different inoculation modes of M-111 against brucellosis infection after exposure to virulent Brucella. Figure 7. funnel chart of the protective effect of different inoculation modes of Rev.1 against brucellosis infection after exposure to virulent Brucella. Figure 8: funnel chart of the protective effect of different inoculation modes of S2 against brucellosis infection after exposure to virulent Brucella. Figure 9: funnel chart of the protective effect of vaccination of different sexes against brucellosis infection after exposure to virulent Brucella. Figure 10: funnel chart of the protective effect of different vaccines against brucellosis infection in females after exposure to virulent Brucella. Figure 11: funnel chart of the protective effect of different vaccines against brucellosis infection in males after exposure to virulent Brucella. Figure 12: funnel chart of the protective effect of different vaccines against brucellosis infection in pregnant sheep after exposure to virulent Brucella. Figure 13: funnel chart of the protective effect of vaccination of sheep of different ages against brucellosis infection after exposure to virulent Brucella. Figure 14: funnel c [file 5524768.f3.docx]

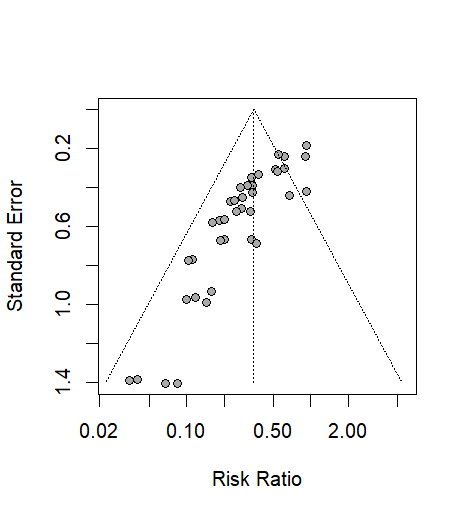


Figure S1. Funnel chart of the protective effect of vaccination with different species against brucellosis infection after exposure to virulent *Brucella*


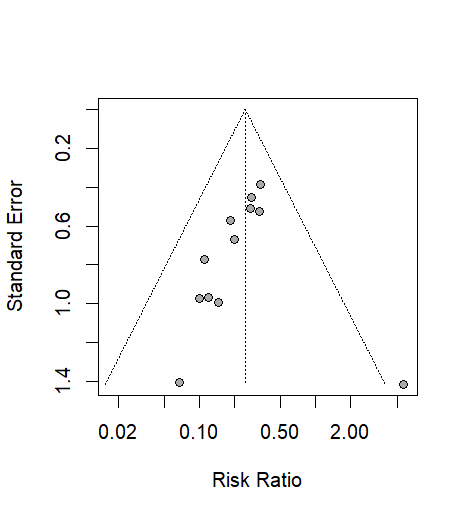


Figure S2. Funnel chart of the protective effect of different doses of M-111 inoculation against brucellosis infection after exposure to virulent *Brucella*


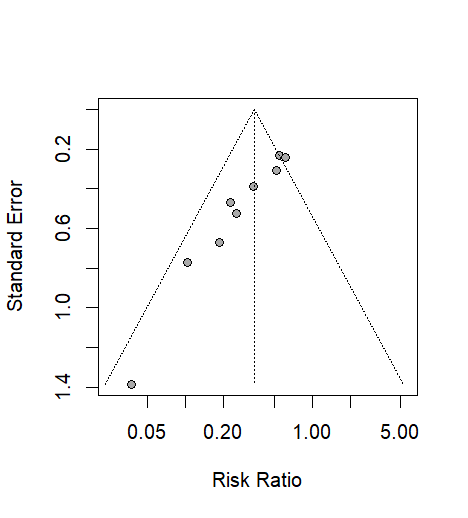


Figure S3. Funnel chart of the protective effect of different doses of Rev.1 inoculation against brucellosis infection after exposure to virulent *Brucella*


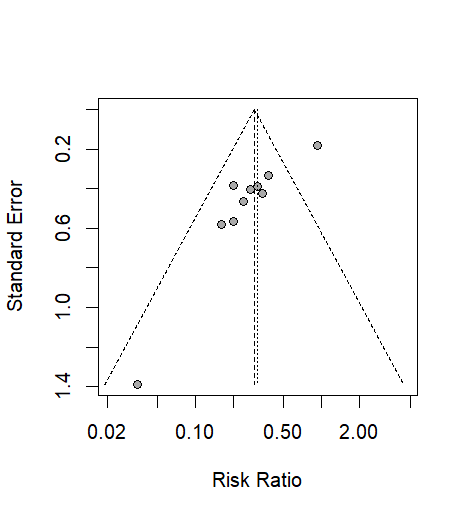


Figure S4. Funnel chart of the protective effect of different doses of S2 inoculation against brucellosis infection after exposure to virulent *Brucella*


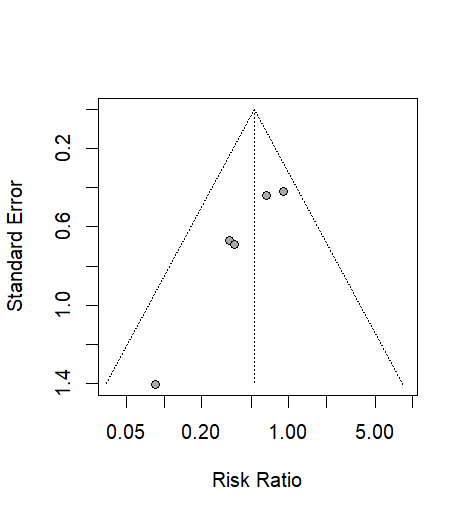


Figure S5. Funnel chart of the protective effect of different doses of B. ovis inoculation against brucellosis infection after exposure to virulent *Brucella*


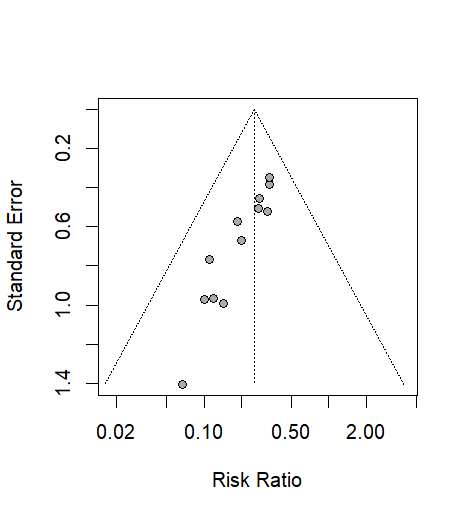


Figure S6. Funnel chart of the protective effect of different inoculation modes of M-111 against brucellosis infection after exposure to virulent *Brucella*


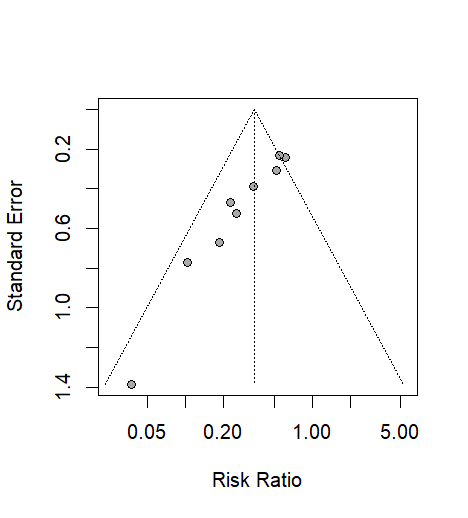


Figure S7. Funnel chart of the protective effect of different inoculation modes of Rev.1 against brucellosis infection after exposure to virulent *Brucella*


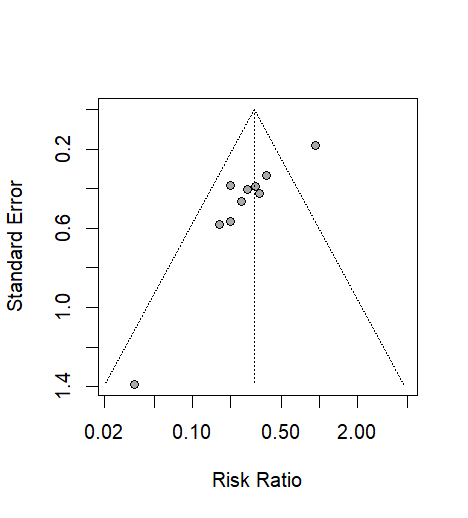


Figure S8. Funnel chart of the protective effect of different inoculation modes of S2 against brucellosis infection after exposure to virulent *Brucella*


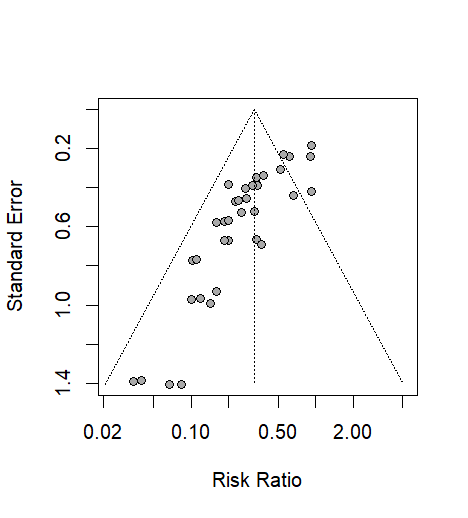


Figure S9. Funnel chart of the protective effect of vaccination at different sex against brucellosis infection after exposure to virulent *Brucella*


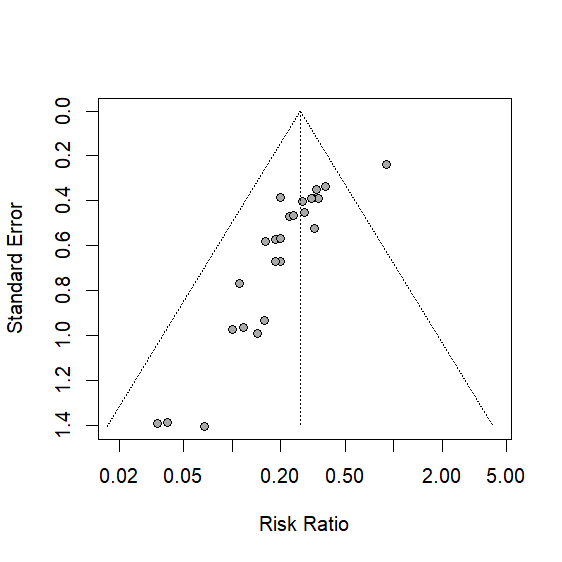


Figure S10. Funnel chart of the protective effect of different vaccines against brucellosis infection in females after exposure to virulent *Brucella*


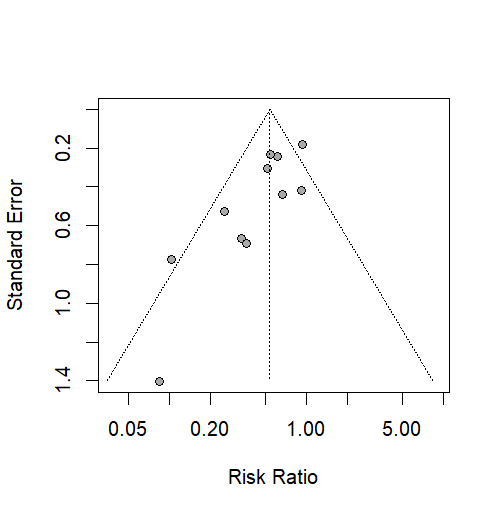


Figure S11. Funnel chart of the protective effect of different vaccines against brucellosis infection in males after exposure to virulent *Brucella*


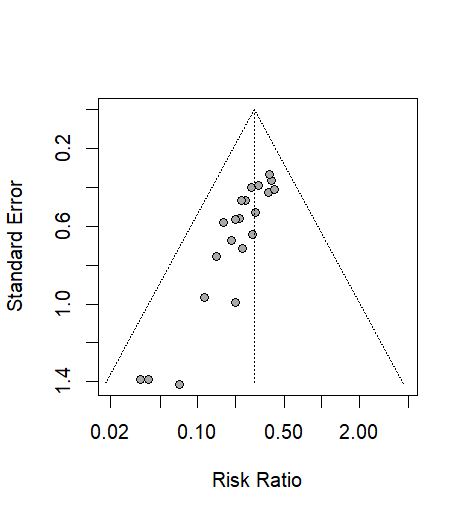


Figure S12. Funnel chart of the protective effect of different vaccines against brucellosis infection in pregnant sheep after exposure to virulent *Brucella*


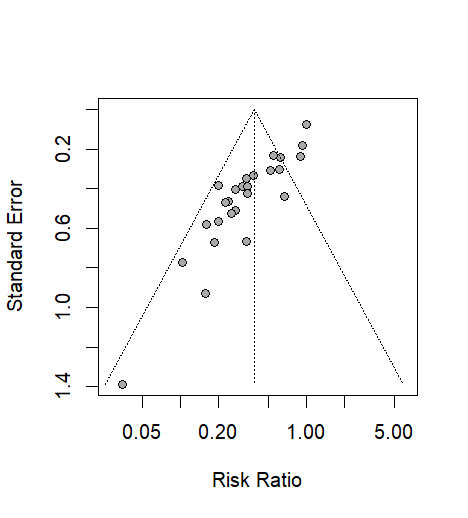


Figure S13. Funnel chart of the protective effect of vaccination of sheep of different ages against brucellosis infection after exposure to virulent *Brucella*


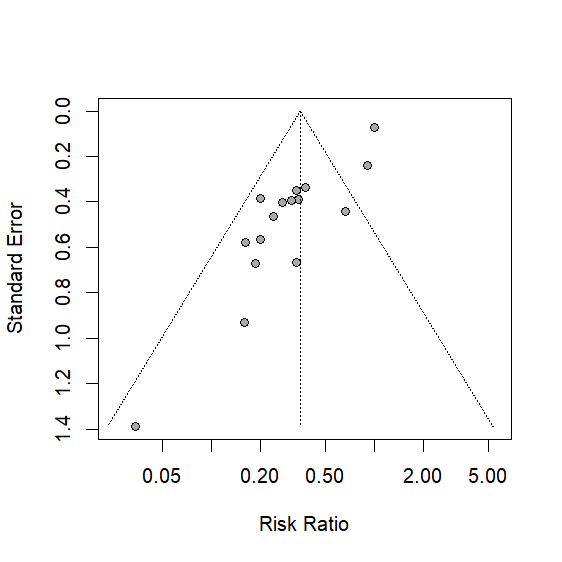


Figure S14. Funnel chart of the protective effect of different vaccines against brucellosis infection in adult sheep after exposure to virulent *Brucella*


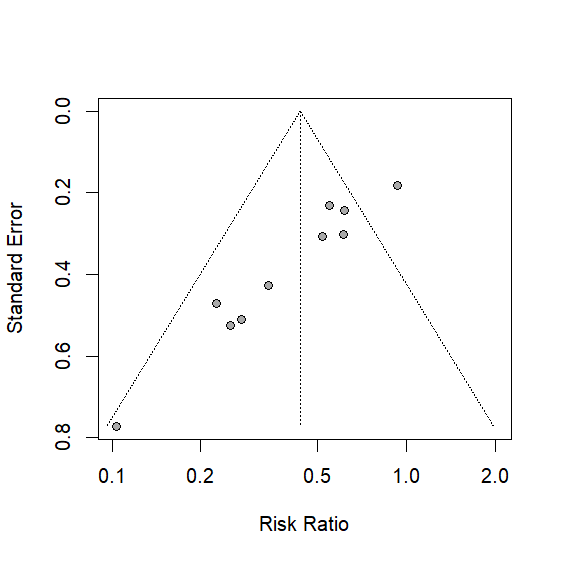


Figure S15. Funnel chart of the protective effect of different vaccines against brucellosis infection in lamb after exposure to virulent *Brucella*


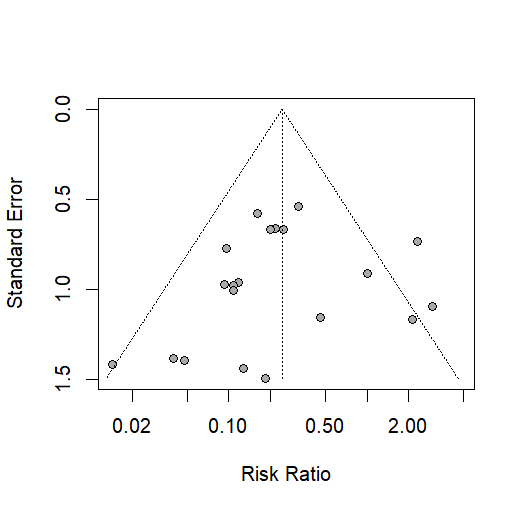


Figure S16. Funnel chart of the protective effect of different vaccinations against clinical signs of brucellosis (miscarriage) following exposure to virulent *Brucella*
